# Editorial: Safety and side effects of psychotropic medications

**DOI:** 10.3389/fpsyt.2023.1148158

**Published:** 2023-02-09

**Authors:** Renato de Filippis, Mireia Solerdelcoll, Mohammadreza Shalbafan

**Affiliations:** ^1^Psychiatry Unit, Department of Health Sciences, University Magna Graecia of Catanzaro, Catanzaro, Italy; ^2^Department of Child and Adolescent Psychiatry, Institute of Psychiatry, Psychology & Neuroscience, King's College London, London, United Kingdom; ^3^Department of Child and Adolescent Psychiatry and Psychology, Institute of Neuroscience, Hospital Clínic de Barcelona, Barcelona, Spain; ^4^Mental Health Research Center, Psychosocial Health Research Institute (PHRI), Department of Psychiatry, School of Medicine, Iran University of Medical Sciences, Tehran, Iran

**Keywords:** adverse drug reaction, adverse effects, mental disorders, mental health, psychiatry, psychopharmacology, real world evidence (RWE), tolerability

In the last few decades psychopharmacology has witnessed an incredible development in terms of efficacy, safety and number of options available for the treatment and management of major mental disorders (1, 2). The change of perspective from a therapy mainly centered on experience to the evidence-based medicine has represented a true Copernicus revolution in mental health, with the implication of providing concrete improvements on disease prognosis and patients' quality of life (3). Despite this, the tolerability profile of psychopharmacological therapy remains an area for improvement on which patients, caregivers, and health care providers agree (4, 5). To fill this gap, various strategies have been applied, which improve the quality of data available on adverse drug reactions (ADRs) related to psychiatric therapy (6–8). Notherless, much remains to be investigated about the systemic effects of psychoactive medicines, their pharmacokinetic and pharmacodynamic interactions, and the variability of these consequences in frail and special populations (9–13).

Therefore, in this Research Topic, we aimed to collect articles dealing with the safety and side effects of psychotropic medications in mental health. We welcomed all articles potentially able to add some elements of novelty and evidence on this sensitive topic.

Several papers focused on the role and safety of antipsychotic medications. Hakami et al. provided a retrospective cohort study investigating the antipsychotics-induced weight gain with metformin co-administration. Retrospectively screening 4,141 medical records of a psychiatric outpatient clinic, authors found a total of 395 patients' records, showing that the concomitant use of metformin was related to reduction in the weight gain tendency.

The paper by Yunusa et al. evaluated the hospitalizations related to serious ADRs associated to atypical antipsychotics (AAPs). In this cross-sectional analysis authors used the FDA Adverse Event Reporting System (FAERS) database (from 2004 to 2021) to examine disproportionality in reporting hospitalizations suspected to be associated with 12 AAPs. According to this analysis, patients taking clozapine, olanzapine, quetiapine, risperidone, aripiprazole, and ziprasidone were more likely to report being hospitalized than users of other AAPs.

Following a similar research path, Peters et al. investigated the cumulative time spent in second generation antipsychotics (SGAs), antipsychotic polypharmacy, and clozapine in discharged patients affected by schizophrenia. Data from 2,997 patients with a minimum of 6 weeks medicated with SGAs were analyzed. Patients suffering from schizophrenia accumulated 44 months in SGA monotherapy, 4 months in polypharmacy, 11 months in medication gaps, and 17 days in clozapine over a 5-year period.

Zhu et al. reported data about 378 long-term hospitalized patients diagnosticated with schizophrenia analyzing the factors influencing comorbid type 2 diabetes mellitus (T2DM) on prolactin levels in their long-term stay in hospital. Surprisingly, compared with patients without T2DM, the patients in the T2DM subgroup had lower prolactin levels and rather severe psychiatric symptoms, with aripiprazole as a protective factor for hyperprolactinemia in long-term hospitalized patients, and female gender representing a risk factor.

The study by Akbarzadeh, Niksun et al. investigated the relevant hypothesis of inflammatory processes in central nervous system in bipolar disorder, through the co-administration of curcumin, with anti-inflammatory effects, and sodium valproate. However, the results of this randomized double-blind trial study did not identify any specific clinical advantages in the group taking curcumin compared to the placebo harm, reducing the weight that this option can play in the management of the tolerability of drug therapy.

One of the included papers in our Research Topic reported a case report describing neutropenia after the coadministration of clozapine and nirmatrelvir-ritonavir in a patient with SARS-CoV-2 infection, adding a literature review on the topic (Liu et al.). Indeed, the risk of neutropenia may increase during SARS-CoV-2 infection and co-administration of clozapine and the antiviral drug paxlovid. Thus, the white blood cell count and absolute neutrophil count should be closely monitored in this specific group of patients.

The paper by Khazaie et al. presented a systematic review and meta-analysis on randomized, double-blind, placebo-controlled trials on suvorexant and lemborexant, dual orexin receptor antagonists for treatment of insomnia. The study includes eight articles (five for suvorexant and three for lemborexant) evaluating diary measures, rating scales, polysomnography results, treatment discontinuation, and ADRs. Overall, efficacy favorably differs in both suvorexant and lemborexant groups compared to placebo. Safety profile did not differ significantly except for somnolence, excessive daytime sleepiness and nightmare in the treatment groups, but without severe ADRs reported. Authors conclude that suvorexant and lemborexant are efficacious and safe agents for insomnia treatment.

The last two papers describe peculiar case reports. The first one, by Akbarzadeh, Behravan et al., portrayed a case of citalopram-induced sleep bruxism in a 9-month-old female breastfed infant whose mother recently used citaliporm (10 mg per day) for her anxiety disorder. Then, patient's bruxism symptoms disappeared following the citalopram discontinuation by her mother.

The other case is reported by Shi et al., describing the treatment of psychiatric symptoms in a 41-year-old male patient suffering from pituitary adenoma with acromegaly since 8 years, more recently associated to untypical neuropsychiatric disturbances. Indeed, he developed an acute psychotic episode, such as to require hospitalization and good response and tolerability to aripiprazole.

All papers described in this Editorial explore several safety and tolerability aspects of medications in mental health. Considering the large utilization of such category and the relative paucity of evidence available about their long-term consequences (14), the articles gathered in this Research Topic shed some light on the clinical implications of the pharmacological approach throughout a wide range of different settings, conditions and samples.

Although research on psychopharmacology has already produced a large amount of data on many aspects (15, 16), we believe that the clinical framework offered in these articles provides a practical and original point of view that could lead to a more targeted and specific use of forces and resources, arousing the curiosity of clinicians and researchers.

Considering the psychopharmacological trends described in the included papers, we highlight the urgent need to deepen psychotropic drugs-related ADRs. The results collected in this Research Topic do not discourage the prescription of psychotropic drugs, but rather aim to promote their conscious use.

## Author contributions

All authors listed have made a substantial, direct, and intellectual contribution to the work and approved it for publication.
